# Visual imitation learning from one-shot demonstration for multi-step robot pick and place tasks

**DOI:** 10.1038/s41598-025-30938-x

**Published:** 2025-12-05

**Authors:** Shuang Lu, Christian Härdtlein, Johaness Schilp

**Affiliations:** 1 Fraunhofer Institute for Casting, Composite and Processing Technology, Am Technologiezentrum 10, Augsburg, 86159 Germany; 2 University of Augsburg, Am Technologiezentrum 8, Augsburg, 86159 Germany

**Keywords:** Engineering, Information technology

## Abstract

Imitation learning provides an intuitive approach for robot programming by enabling robots to learn directly from human demonstrations. While recent visual imitation learning methods have shown promise, they often depend on large datasets, which limits their applicability in manufacturing scenarios where tasks and objects are highly specialized. This paper proposes a one-shot visual imitation learning framework that allows robots to acquire multi-step pick & place tasks from a single video demonstration. The framework integrates hand detection, object detection, trajectory segmentation, and skill learning through Dynamic Movement Primitives (DMPs). Hand trajectories are mapped to the robot’s end-effector, enabling the system to generalize to new object positions while significantly reducing data requirements. The approach is evaluated in simulation and achieves reliable reproduction of multi-step tasks. These results demonstrate the potential of one-shot visual imitation learning to reduce programming complexity and increase flexibility for industrial robot applications.

## Introduction

Recent changes in manufacturing, such as increasing product differentiation, personalization, and high-mix, low-volume production, have made flexibility a central requirement for industrial robotics^1^. Pick & place systems, in particular, must be able to handle objects of varying shapes, sizes, and weights, and adapt quickly to changing production demands. Traditional robot programming approaches are not well suited for such environments, as they require expert knowledge, are time-consuming, and lack scalability^2,3^.

Learning from demonstration offers an intuitive alternative, enabling robots to learn tasks directly from human demonstrations. This approach is accessible to non-expert users and has been explored through both physical guidance of robot manipulators^4^ and visual observation using cameras^5^. Advances in computer vision have made visual imitation learning especially attractive, as it decouples the task model from robot-specific physics while leveraging visual data to capture human actions.

Most existing visual imitation learning research has focused on household applications. In these domains, large datasets and virtual environments are readily available, supporting the development of deep learning models that map visual inputs to robot actions^6–10^. However, manufacturing scenarios differ significantly: tasks are more diverse, objects are domain-specific, and standardized datasets are scarce. Creating large-scale training environments for each use case is inefficient and often impractical. This creates a need for efficient approaches that reduce data requirements.

Recent advances such as VIEW and K-VIL have made significant progress in visual imitation learning. VIEW^11^ improves sample efficiency by condensing human trajectories into waypoints and accelerating learning with additional rollouts, but it remains a few-shot approach that requires exploration beyond a single demonstration. K-VIL^12^ builds keypoint-based geometric constraints from videos to achieve viewpoint- and embodiment-invariant task representations. While effective in household and lab settings, it relies on extracting keypoints directly from demonstrations and does not address the lack of labeled training data for domain-specific industrial objects. In contrast, this work focuses on one-shot learning, where a single video demonstration is sufficient to reproduce multi-step tasks, and integrates CAD-based synthetic data generation for object detection. This design leverages the availability of CAD models in manufacturing while ensuring robust segmentation into *Reach*, *Grasp*, *Move*, and *Release* skills.

The main contribution of this work is a one-shot visual imitation learning framework for multi-step pick & place tasks in manufacturing. Unlike prior few-shot approaches, the proposed framework learns from a single video demonstration without additional rollouts. It integrates four components: 1) hand detection, 2) CAD-based synthetic data–driven object detection, 3) trajectory segmentation with object-state refinement, and 4) skill learning through Dynamic Movement Primitives (DMPs) into a coherent and modular pipeline. This design reduces the need for manual data annotation, ensures robust identification of grasp and release events, and enables trajectory generalization to new task configurations.

## Related work

In order to understand and evaluate the results of visual imitation learning, the learned outcomes are commonly categorized into three levels: the skill level, the task level, and the goal level. An overview of these abstraction levels, together with illustrative examples, is provided in Table 1.

**Table 1.**
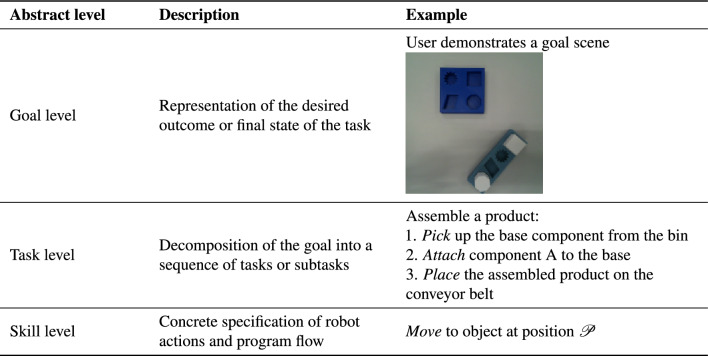
Hierarchical abstraction levels for robot programming.

### Skill level

Finn et al. presented a visual imitation learning method that enables a robot to learn new skills such as *push* and *place* from raw pixel input^6^. The policy is represented by Convolutional Neural Networks (CNN). The policy observation includes both the RGB image and the robot’s joint angles and end-effector pose. A policy $\pi$ is learned to map observations to robot actions. The approach integrates meta-learning with imitation learning, allowing a robot to reuse experience and quickly learn new skills from a single demonstration. This is achieved through a two-level learning process: the inner loop makes task-specific adjustments, while the outer loop updates meta-parameters across tasks. However, the two-level training process is complex, and the learned model is adapting poorly to environment changes. Xin et al. developed a so-called IRMT-Net to predict the interaction region and motion trajectory from RGB-D images^9^. The input of the developed model consist of motion trajectories, motion category and cropped object images. To generate motion trajectory from RGB-D images, the hand detection method proposed by Shan et al.^13^ is applied to extract bounding box from each RGB frame. The center coordinate of the bounding box and its depth value are taken as 3D hand coordinates. Faster-RCNN^14^ is used to detect objects. The cropped image for the detected object is obtained based on the bounding box with the highest detection score. To evaluate the proposed approach, a dataset is created by labeling the motion trajectories for videos in the Epic-kitchens dataset^7^ with 9236 videos. In the end, skills such as *pull open drawer* and *take cup* in kitchen scenarios are learned. The annotated dataset contributes to the further development of visual imitation learning for kitchen scenarios. Wen et al. introduced a method for learning task trajectories using a single demonstration video, captured by a statically mounted Photoneo 3D camera^15^. This camera records at 10 Hz. It provides gray-scale and depth images, allowing for detailed observation of the working environment. The method involves tracking target objects to generate trajectories, which demonstrated effective performance. A key factor contributing to the success of this approach is the use of relatively large objects, which minimizes occlusion by the human hand during the demonstration.

### Task level

Qiu et al. presented a system with observing human demonstrations by an ASUS RGB-D camera^5^. During demonstration, a human worker performs an object handling task wearing a colored hand glove. This hand glove improves the accuracy and robustness for hand detection. The hand pose is estimated based on a deep learning model trained by 3D input data^16^. The human demonstration is segmented by Hidden Markov Models (HMMs) into motion primitives called skills. They include *pick up*, *place* and *locate*. The skills are then represented by Dynamic Movement Primitives (DMPs)^17^, which allows the generalization to new goal positions. Notably, there are no consensus for defining the semantic of skills in the existing works. Qiu et al. consider *pick up*, *place* and *locate* as skills^5^. However, Kyrarini et al. define them as *start arm moving*, *object grasp*, *object release*^18^. This discrepancy in definitions presents a challenge when comparing the performance of different approaches.

### Goal level

Zeng et al. proposed an approach to enable robots to understand and execute tasks by interpreting the goals from demonstrations provided by the users^19^. The initial and goal scene are represented by RGB-D images. A method called Discriminatively-Informed Generative Estimation of Scenes and Transforms (DIGEST) was developed to generate scene graph from demonstrated images. The first step in the DIGEST method involves detecting bounding boxes in the RGB images. Once objects are detected, their pose is estimated using the depth information from the RGB-D images and existing object mesh models. The final step is to generate a scene graph. This graph represents the spatial and relational structure of the scene. It is built using inter-object relations such as *exist*, *clear*, *in*, and *on* by calculating the object poses. Given the observation of the goal state of the world, the robot estimates the goal scene graph, and stores the desired inter-object relations by PDDL^20^. It is a formal language used for expressing planning problems and domains. The task planner gives a sequence of high-level pick & place actions. To pick an object, the robot receives a number of pre-computed grasping positions for the object, and uses Moveit! to determine which of these positions can generate a collision-free path.

### Summary

In conclusion, visual imitation learning leverages the latest advancements in computer vision and machine learning to enhance robot programming at multiple levels. These levels include the execution of basic skills, the understanding of complex task sequences, and the achievement of task goals. For multi-step pick & place tasks, this work implements the imitation learning at the task level, focusing on the integration and sequencing of skills to perform complex, multi-step tasks. Focusing solely on the goal level risks losing important procedural information, while learning at the skill level alone is insufficient for handling multp-step tasks. As introduced in the first section, this framework is realized by mapping the hand trajectories to the robot’s end-effector. Comparing to the approach of Wen et al.^15^, tracking the hand is also necessary as small objects may be occluded and not visible. In this work, both hand and object trajectories are employed to generate a high-level task plan and low-level trajectories.

## Methods

### Task model representation

**Fig. 1:**
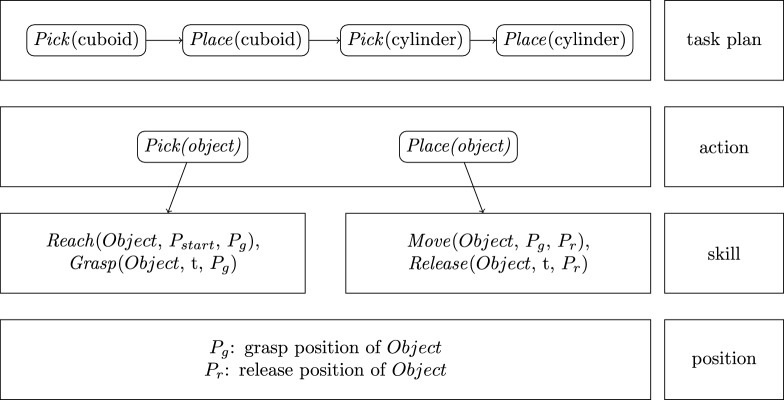
Illustration of the hierarchical task model, representing the decomposition from task-level planning to skill execution and final position specification.

To address the three variations mentioned above, a task model is defined by modifying the approach of Backhaus, which was developed for adaptive programming system for assembly tasks^21^. The modified task model consists of four layers, as illustrated in Fig. 1. It listed from high-level task plan to low-level position. The task plan is a sequence of actions to complete the task. Each action has a parameter of an object. A *pick* action refers to the act of selecting and lifting an object from a particular location. It involves grasping the object to obtain control over it. The *pick* action is further divided into *Reach* and *Grasp* skills. *Reach* is represented by motion from random start to grasp position. *Grasp* is considered as a discrete event, which occurs in single timestamp. It represents the moment when robot’s end-effector successfully makes contact with the object and securely holds it. The *place* action is divided into *Move* and *Release* skills. *Move* is represented by motion from grasp to release positions with the moving object. *Release* is also considered as a discrete event. It involves opening the gripper from the end effector. Among these skills, *grasp* and *release* are the most critical, as they mark the transitions between motion phases and directly determine the success of the overall task. In this work, these skills are extracted from the demonstration video. The estimation of precise grasp and release poses from visual data is not in the scope of this study. Instead, we note that pose estimation and 6D object alignment are addressed by benchmarks such as the BOP challenge^22^, which could be incorporated in future work. This structured task model provides a clear hierarchical representation that helps the robot to effectively understand and execute tasks in dynamic environments.

### Hand detection

There are several trained models and frameworks available for hand detection. In this work, two common approaches used in the context of visual imitation learning, one from the Bbox-based approaches and one from the keypoints-based approach are selected to evaluate hand movements. As discussed in the second section, Xin et al. applied a pre-trained Bbox-based model to learn hand motion trajectories with promising results^9^. The model was proposed by Shan et al. to understand hand and hand-object interaction^13^. The system was built on top of a popular object detection system, Faster-RCNN^14^. The model has a two-stage architecture, where the first stage proposes regions and the second stage classifies and refines the bounding boxes. The models were trained on the datasets proposed in the same publication, the 100DOH dataset and the 100 K frames. The 100DOH is a large video dataset containing hands and hand-object interactions. They are 27.3K Youtube videos from 11 categories with nearly 131 days of footage of everyday interaction. To detect hand on single image, the authors created a new 100 K frame-level dataset, which consists of 99,899 frames extracted from the videos in 100DOH and VLOG Dataset^23^. In total, there are 189,426 annotated bounding boxes for hand. Two models trained on 100 K and 100K+ego are provided by the authors for hand detection. The 100K+ego consists of 56.4K frame subset of egocentric data from^10,24,25^. Both models achieve approximately 90% average precision.

**Fig. 2:**
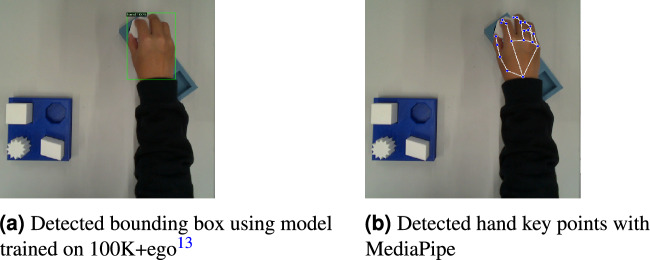
Results of Hand Detection on RGB Images Using Different Models.

Another detection method is based on keypoints, which is an open-source framework developed by Google called MediaPipe^26^. It provides a pipeline for hand detection. The output of the hand detector are 21 3D hand-knuckle coordinates inside the detected hand regions. The representation of each keypoint is composed of *x*-, *y*- and *z*- coordinate, where *x* and *y* are pixel coordinates on image and *z* is the distance to the wrist as origin. A hand trajectory can be created from either method using the center of the hand position. The examples for both methods are illustrated in Fig. 2a and b. The two methods will be evaluated in section 4.

### Object detection

**Fig. 3:**
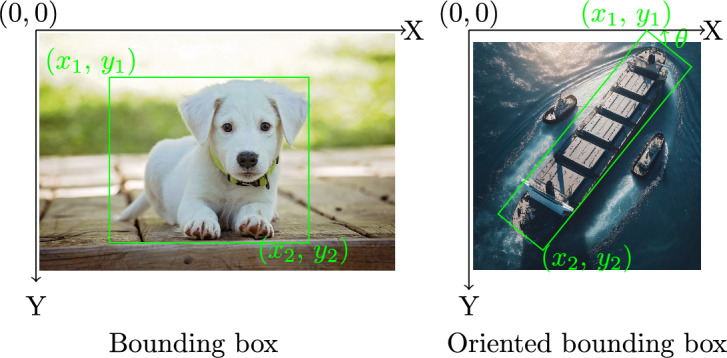
Illustration of the differences between axis-aligned Bounding Box (Bbox) and Oriented Bounding Box (OBB) annotation.

As discussed in the second section, object detection in visual imitation learning is crucial for understanding task environments. Thanks to the availability of large datasets, various model architectures have been developed and shown to continuously improvement performance. Most of available datasets are labeled with regular bounding boxes for everyday objects. They serve as benchmarks for comparing different model architectures. Some well known datasets are ImageNet^27^, Pascal VOC^28^ and COCO^29^. They provide raw images, its annotations and standardized evaluation protocols. COCO is currently the standard benchmarking dataset for the object detection community. The latest release of COCO 2017 consists of a total of 123,287 images and 896,782 objects, across its validation datasets. Additionally, the test dataset comprises 40,670 images. They cover 80 object categories, which include everyday objects such as person, bicycle, and car, as well as household items like chair, couch, and dining table. The annotation include the bounding box coordinates, the category level, and other optional attributes. The bounding boxes provided in COCO are rectangular boxes aligned with the image exes that enclose the object. They do not account for the object’s rotation. However, the orientation is essential for application scenarios such as understanding aerial images. Dataset of Object deTection in Aerial images (DOTA)^30^ is a large-scale dataset for the application of aerial images. The DOTA dataset consists of a total of 1,793,658 object instances that are annotated with oriented bounding box (OBB) annotations. The illustration of the annotations is shown in Fig. 3, where $\theta$ refers to the rotation of the rectangular box with respect the image axis. The object instances in DOTA belong to 18 different categories, which include objects like planes, ships, and basketball courts. The OBB approach is followed in this work, which allows for improved accuracy and better object differentiation.

As discussed above, existing datasets primarily cover everyday objects. To develop object detection models for a specific task in manufacturing scenarios, it is necessary to generate a specific set of objects. The data generation process involves capturing images and manually annotating the objects of interest, which is time-consuming. CAD files are usually available in manufacturing scenarios. Synthetic training data can be generated using CAD data and has gained popularity in recent years due to its advantages in providing accurately labeled datasets at a lower cost^31^.

Several platforms support synthetic data generation, including Blender, Unity, and NVIDIA Omniverse Replicator^32^, all of which have demonstrated strong performance in object detection tasks^33,35,35^. Each platform has trade-offs: Blender provides high flexibility and open-source availability but may require more scripting effort and slower rendering for large-scale datasets; Unity offers user-friendly tools and a rich asset ecosystem but less control over low-level rendering and potential licensing constraints; and Omniverse enables photorealistic rendering and advanced sensor simulation but demands significant computational resources and has a steeper learning curve. In this work, Blender was selected because it is open-source, highly flexible, and well established in academic research. It allows full control over rendering, annotation, and domain randomization, while integrating smoothly into automated pipelines. The authors’ prior expertise with Blender further enabled efficient customization and rapid adaptation to the manufacturing scenario. The details of the synthetic data generation process are discussed below.

#### Data generation

**Fig. 4:**
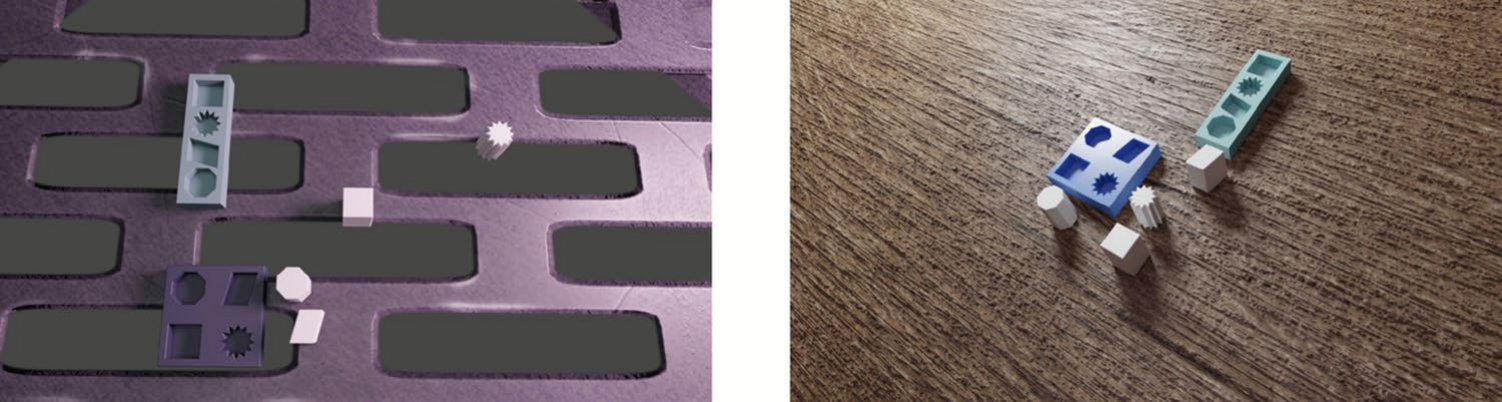
Generated photorealistic images with Blender.

Photorealistic images are created or rendered to closely resemble real-life objects or scenes. Hodaň et al. developed an approach to synthesize photorealistic images of 3D models, which are used to train a convolutional neural network for detecting the objects in real images^22^. The approach is implemented in BlenderProc, which is an open source pipeline to render the images^35^. The pipeline is a Blender extension with Python API with various examples. The image synthesis approach for generating LineMOD dataset in BOP challenge is followed in this work. In this approach, objects are arranged inside an empty room. A random photorealistic material from the CC0 Textures library is assigned to the walls of the room, and light with a random strength and color is emitted from the room ceiling and from a randomly positioned point light source. Realistic object poses are achieved by dropping objects on the ground plane using PyBullet physics engine integrated in Blender. In the pipeline, both the images and their corresponding annotations are produced simultaneously and automatically. The annotations are generated in OBB format to preserve the orientation of the objects. Examples of photorealistic images of 6 CAD models are shown in Fig. 4, which are grayBox, blueBox, cuboid, parallelogram, star and octagon.

#### Fine-tuning

YOLOv8 is utilized in this work to detect object of interests due to the availability of pre-trained models for OBB detection and its software framework. The model utilizes a convolutional neural network that can be divided into two main parts: backbone and detection head. The head of YOLOv8 consists of multiple convolutional layers followed by a series of fully connected layers. These layers are responsible for predicting the oriented bounding boxes and class probabilities for the objects detected in the image. During fine-tuning, the parameters from the model backbone are used to initialize the model, the detection head is initialized with number of classes for the new task. The fine-tuning of the YOLOv8 model with the photorealistic images and the validation on real images will be discussed in the fourth section. While OBBs are sufficient for object localization and segmentation, they are not accurate enough to directly estimate grasp and release positions. Full 6-DoF pose estimation is out of scope and left for future work.

### Mapping motion from a human hand to a robot end-effector

**Fig. 5:**
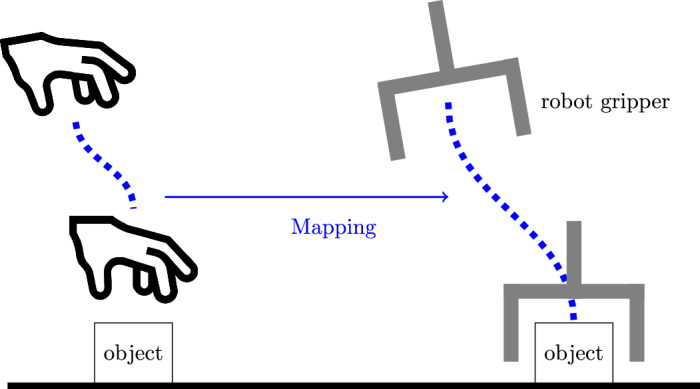
Mapping *reach* motion from a human hand to a robot end-effector.

This section focuses on mapping motion from a human hand to a robot end-effector. Figure 5 illustrates the mapping concept. The framework starts by generating hand and object trajectories from continuous frame-by-frame hand and object detection. These trajectories are then segmented based on the interaction of the hand and object and the object state. The resulting hand motion segments *reach* and *move* are represented as skills with DMPs. These representations are stored in the task model to reproduce the task.

#### Trajectory generation for the human hand and the objects

**Fig. 6:**
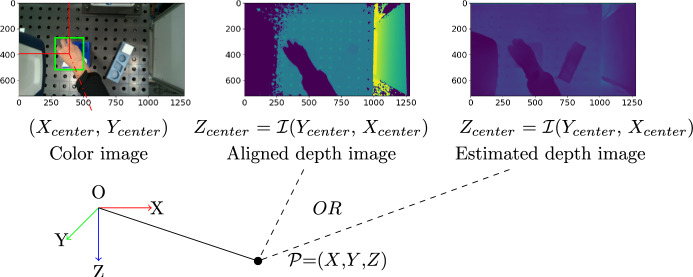
Generating trajectories by transforming hand and object positions from pixel coordinates to world coordinates in the camera frame.

As mentioned in the first section, an RGB-D camera is required to record the visual demonstration. Hand and object detection are performed on the RGB frames. The detected results are bounding boxes at pixel coordinates. The corresponding depth values can be read from the depth frame. The Equation 1 shows the process. The center of detected bounding boxes from RGB images for the human hand and objects are $X_{center}$ and $Y_{center}$. The responding depth value $Z_{center}$ can be identified from depth image. Similar as RGB image, the depth image is represented by a two-dimensional matrix, $\mathscr {I}$ = (*y*, *x*), the coordinate (*x*, *y*) corresponds to the column and row indices, respectively. The depth value $Z_{center}$ can be identified from depth image $\mathscr {I}$($Y_{center}$, $X_{center}$). The resulted sequence of $\mathscr {P}$ = {$X_{center}$, $Y_{center}$, $Z_{center}$} are hand and object trajectories in pixel coordinates as shown in Fig. 6. In the next step, the position is transformed to real world coordinates with camera as origin. In the equation, $f_{x}$ and $f_{y}$ describe the focal length of the image, and the *ppx* and *ppy* describe the pixel coordinates of the principal point. They are camera intrinsic parameters to describe the internal characteristics of the camera.

1 $$\begin{aligned} \begin{aligned} X&= \frac{X_{center} - ppx}{f_{x} \cdot Z_{center}} \\ Y&= \frac{Y_{center} - ppy}{f_{y} \cdot Z_{center}} \\ Z&= Z_{center} \\ \end{aligned} \end{aligned}$$

According to Ghidoni^36^, several technologies are available for estimating depth information: stereoscope, infrared light and LiDAR. A stereoscopic camera captures two images simultaneously from different angles to determine distance. Infrared and LiDAR technologies work on a similar principle. They use a light source and a receiver to measure the time it takes the light to reach the sensor. Both of these technologies are referred to as ToF sensors. Each technique can be affected by lighting conditions and the texture of objects, and have invalid depth values. To overcome bad depth value, an additional estimated depth map from the color image is used in this work. It uses the global-local path networks model for monocular depth estimation^37^. The model is made available by Hugging Face. In case the depth value is unavailable in aligned depth frame from camera, it will be estimated from color frame using the pre-trained model from Hugging Face. The transformation from pixel coordinates to camera frame is also performed using Equation 1.

By repeating the aforementioned process for each pair of RGB and depth frames, the hand and object trajectories can be generated in 3D space. Normally, the object detection accuracy cannot reach $100\%$. If the hand and the object of interest cannot be detected in some frames, the last valid detection is propagated to the next frame. The generated hand and object trajectories can be represented as *Traj* = ($\mathscr {P}_{1}$, $\cdots$ $\mathscr {P}_{i}$, $\cdots$, $\mathscr {P}_{T}$).

#### Motion representation for a robot’s end-effector

**Fig. 7:**
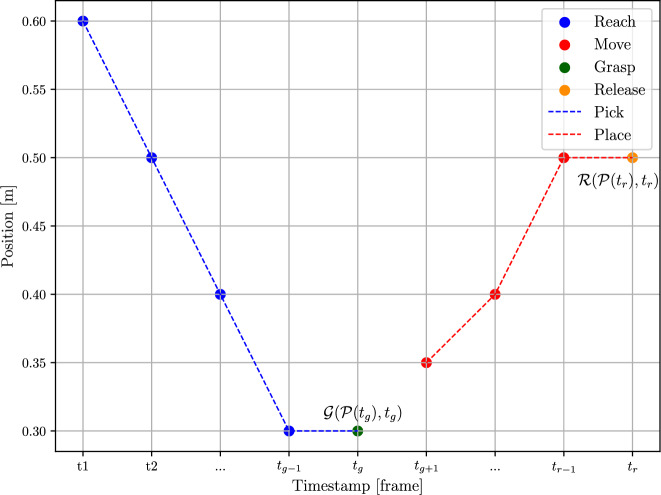
Illustration of the segmented trajectories.

To map hand motions to the robot’s end-effector, the hand trajectories are segmented into smaller components which allows adapting hand to end-effector position. The required small components are *pick* and *place* actions and the *reach*, *grasp*, *move* and *release* skills, as defined in section 3.1. The skills’ representation for the robot’s end-effector during the pick & place process is summarized in Table 2, where $\mathscr {P}(t)$ denotes the position of the end-effector at timestamp *t*. The action of picking up an object involves the *reach* and *grasp* skills. The *reach* skill describes the motion of the end-effector from a starting point to the grasping position. Once the end-effector reaches the object, the *grasp* occurs at the next timestamp. After grasping the object, the *move* skill is used to transport the object to the desired location. Unlike the separate motions of moving and positioning in human hands, transporting an object with a robot is considered as continuous motion. Upon arriving at the desired position, the *release* skill is triggered to release control of the object. An example of the segmented trajectories are illustrated in Fig. 7.

**Table 2:** Representation of motion as skills for robots.

Actions	Skills	Segmented hand trajectories
Pick	*Reach*	$[\mathscr {P}(t_{1}), \mathscr {P}(t_{2}), \dots , \mathscr {P}(t_{g-1})]$
	*Grasp*	$\mathscr {G}(\mathscr {P}(t_{g}), t_{g})$,$\mathscr {P}(t_{g-1}) = \mathscr {P}(t_{g})$
Place	*Move*	$[\mathscr {P}(t_{g+1}), \mathscr {P}(t_{g+2}), \dots , \mathscr {P}(t_{r-1})]$
	*Release*	$\mathscr {R}(\mathscr {P}(t_{r}), t_{r})$,$\mathscr {P}(t_{r-1}) = \mathscr {P}(t_{r})$

**Fig. 8:**
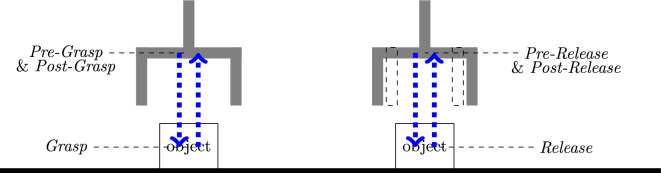
Illustration of Pre-Grasp, Post-Grasp, Pre-Release, Post-Release Phases.

In the practice of robot programming, after defining the grasp and release positions, additional pre- and post-motion segments are created to ensure a controlled grasp and release. The pre-grasp involves approaching the object at reduced speed and corrected orientation. Once the robot reaches the pre-grasp pose, it performs the *grasp* to securely hold the object. After successfully grasping the object, the robot moves back to post-grasp pose with the reversed approach trajectory to avoid collision with the environment. The pre- and post-grasp pose are typically the same, ensuring consistency in the robot’s pose. Once in the pose-grasp pose, the robot transitions to the *move* skill to transport the object to the desired place position. The pre-release involves preparing the robot and the object for release. It includes adjusting the robot’s pose to ensure a controlled release. The speed of pre-release and pose-release is usually reduced compared to the transport motion. After releasing the object, the robot returns to post-release pose to start the next movements. Similar to the pre- and pose-grasp poses, the pre- and post-release poses are typically the same for consistency, as shown in Fig. 8. In summary, to map a human hand trajectory to a robot’s end effector, the hand trajectories are first segmented in to *reach*, *grasp*, *move* and *release*, and then augmented with *pre-grasp*, *post-grasp*, *pre-release* and *post-release*. The orientation $\mathscr {O}(t)$ of the objects is determined during the *pre-grasp* and *pre-release* phases and remains constant throughout the *post-grasp* and *post-release* phases.

$$\begin{aligned}&\mathscr {P}_{\textit{pre-grasp}} = \mathscr {P}_{\textit{post-grasp}} = \mathscr {P}(t_{g}) \pm \Delta p\\&\mathscr {O}_{\textit{pre-grasp}} = \mathscr {O}_{\textit{post-grasp}} = \mathscr {O}(object, t_{g})\\&\mathscr {P}_{\textit{pre-release}} = \mathscr {P}_{\textit{post-release}} = \mathscr {P}(t_{r}) \pm \Delta p\\&\mathscr {O}_{\textit{pre-release}} = \mathscr {O}_{\textit{post-release}} = \mathscr {O}(object, t_{r}) \end{aligned}$$

#### Trajectory segmentation

This subsection explains how hand trajectories are segmented into smaller elements to represent the skills of *Reach*, *Grasp*, *Move*, and *Release*. This is accomplished by identifying the timestamps for grasping and releasing. The input data consists of the hand and object trajectories generated in section 3.4.1. Segmenting hand trajectories directly can be a challenging task. Because hand movements often transition smoothly from one phase to another without clear and distinct boundaries. It is therefore difficult to identify where one segment ends and another begins. To address this issue, an approach is proposed that leverages the status of hand-object interaction and object status. Object states describe whether an object is static or dynamic. For static objects, the results are refined by selecting the OBB with the highest confidence score.

As discussed in section 3.4.1, before segmenting the trajectories, missing values caused by detection failure are filled by propagating the last valid value to the next one. Then, a Kalman filter is applied to reduce noise. The hand trajectories are first segmented into pick and place cycles for each object by identifying the release timestamps. They are achieved by segmenting the object trajectories by Gaussian Mixture Model (GMM). It is a statistical model that represents a probability distribution *p*(*x*, *y*) between input *x* and output *y* variables as a weighted sum of Gaussian distributions.

2 $$\begin{aligned} p(\mathscr {X},y|\theta ) = \sum _{k=1}^{K}p(w_{k})p(\mathscr {X},y|\mu _{k},\Sigma _{k}) = \sum _{k=1}^{K}\pi _{k}\mathscr {N}(\mathscr {X},y|\mu _{k},\Sigma _{k}), \end{aligned}$$

where each Gaussian component $\mathscr {N}(x,y|\mu _{k},\Sigma _{k})$ has prior probability $p(w_{k})$ = $\pi _{k}$, mean $\mu _{k}$, and covariance matrix $\Sigma _{k}$. GMM parameters $\theta$ = $\pi _{k}, \mu _{k}, \Sigma _{k}$ are learned from training data using the expectation-maximization. It can be utilized for motion segmentation by modeling the distribution of trajectories over time. In a pick & place task, the object trajectory can be divided into three distinct parts, the static state before picking, the moving state, and the static state after placing. The first timestamp in the state after placing indicating the release of the hand. In this work, the object trajectories are segmented by the GMM model with *K* = 3 and $\mathscr {X}$ = *Traj* = {$\mathscr {P}_{1}, \cdots , \mathscr {P}_{i}, \cdots , \mathscr {P}_{T}$}.

In the second step, the *grasp* timestamps are used to segment the pick and place motion into *reach* and *move*. When a human hand and other objects are present in the same visual frame, occlusion can occur, which means the hand might partially or fully block the view of the object. This can result in inaccurate object trajectories that do not accurately represent the grasping process. However, the object can be correctly detected at the beginning and end of the demonstration, where the human demonstration has finished or not yet started. The *grasp* timestamp is then determined by calculating the shortest distance between the hand and the object’s initial position. The results are *grasp* and *release* index in the time sequence for each object. The resulted sequence symbols for three objects can be represented as [*Reach*($O_{1}$), *Grasp*($O_{1}$), *Move*($O_{1}$), *Release*($O_{1}$), *Reach*($O_{2}$), *Grasp*($O_{2}$), *Move*($O_{2}$), *Release*($O_{2}$), *Reach*($O_{3}$), *Grasp*($O_{3}$), *Move*($O_{3}$), *Release*($O_{3}$)].

The segmentation approach is summarized in Algorithm 1.

**Algorithm 1:**
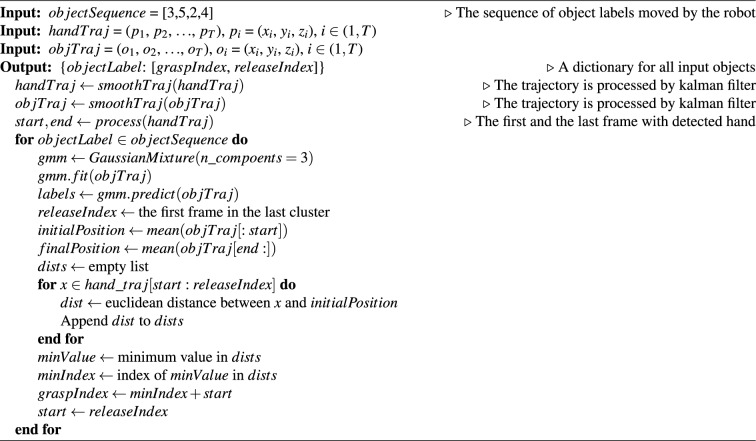
Segmentation hand trajectories

#### Trajectory learning & generation

DMP can learn from a single demonstration and adapt to new start and goal positions^18^. They are therefore used to model goal-directed behaviors. The resulting *reach* and *move* segments are represented by DMPs, which are formalized as stable nonlinear attractor systems^17^. There are many variations of DMPs. As summarized by Fabisch^38^, they have in common that

- they have an internal time variable (phase), which is defined by a so-called canonical system,
- they can be adapted by tuning the weights of a forcing term and
- a transformation system generates goal-directed accelerations.

**Fig. 9:**
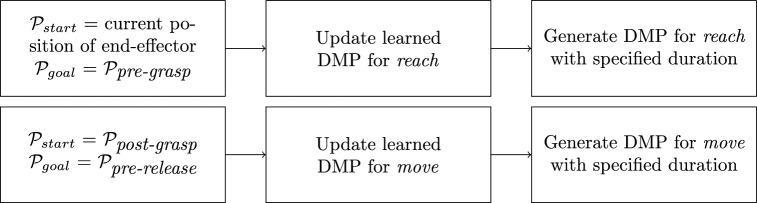
Trajectory generation process.

The formulation of DMPs can be found in^38,40,40^. The choice of coordinates for representing a model can make a big difference in how the generalization of the dynamics systems appears^39^. The DMPs are represented in the camera frame in both the trajectory learning and generation processes. Figure 9 illustrates the steps for generating robot end-effector trajectories using DMP. To generate the trajectory for *reach*, the process begins with defining the current position of the end-effector and aims to reach the pre-grasp position. The learned DMPs from the hand trajectories are updated based on defined initial and goal position of the target object. This process generates a smooth trajectory for the specified duration. Similarly, DMP for *move* is updated by defining the post-grasp position and the pre-release position. The adaptability of DMP allows for updating with new initial and goal position, ensuring controlled movements in dynamic environment.

## Experimental results

### Experimental setup

**Fig. 10:**
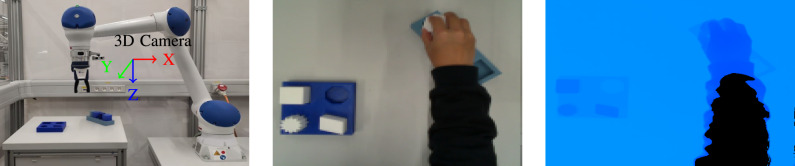
Overview of the demonstration setup and recorded RGB-D data. (**a**) Demonstration setup; (**b**) Color image; (**c**) Aligned depth image.

The goal of this work is to extract the required information from a single video. To avoid any potential bias or singular occurrences, five sets of instructions and their corresponding videos were recorded and evaluated. Figure 10a illustrates the experimental setup to record the videos. The Intel^®^ RealSense^TM^ L515 3D camera is mounted on the robot to record the demonstration process. The camera is positioned to capture the task environment and hand movements. The task environment is shown in Fig. 10b. As a LiDAR camera, it projects an infrared laser at 860 nm wavelength as an active light source. 3D data is obtained by evaluating the time required for the projected signal to bounce off the objects in the scene and return to the camera^41^. The frame rate is 30 frames per second (FPS). The videos were recorded using Intel RealSense Viewer and saved as bag files which can be extracted as a sequence of color and depth images. The size of the color images recorded by the L515 is $1280$ $\times$ $720$ and the depth image size is $640$ $\times$ $480$. The depth frame is rescaled and aligned to the color frame (see Fig. 10c), so that the depth value can be read by the pixel coordinates of the color image.

The videos feature the same four pick & place tasks, which involve transporting a cuboid, star, parallelogram, and octagon from a blue box to a gray box. At the beginning and end of the recording, the task environment was observed without the presence of hands, ensuring a clear view of the working environment for accurate analysis of the conditions before and after manipulation was performed. The task was performed with one hand only.

### Evaluation of hand detection methods

**Fig. 11:**
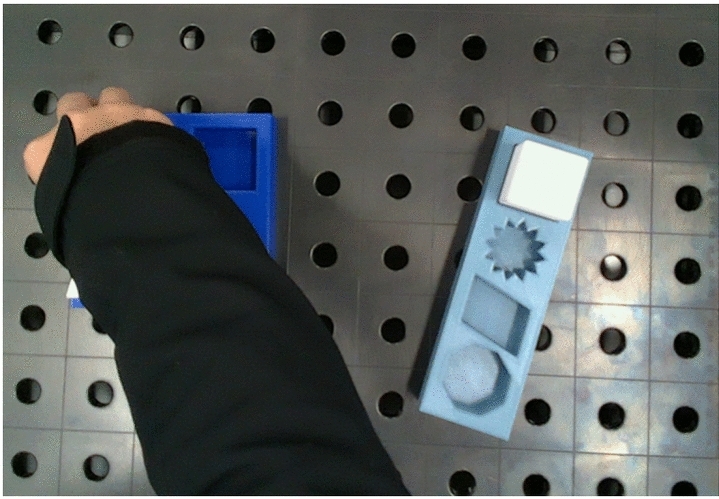
An instance of missed hand detection by the model Faster-RCNN.

The models provided by Shan et al.^13^ are available online as open source. The detection process is integrated into Detectron2, which is a framework for computer vision tasks such as object detection and instance segmentation. The model trained on 100 K + ego was used to run the test. The score threshold for filtering out low-confidence detection was set at 0.7. Any model output below this threshold is considered as invalid. A low threshold means that the model accepts detections with lower confidence levels, which can significantly increase the number of false positives. The performance of the both methods are evaluated on the five recorded videos, which consists of 3601 images in total, where hand are visible in 2631 images. The results are summarized in Table 3. The percentages in the table represent the proportion of images where the methods successfully detected hands when hands are present. It was observed that the model failed when the hand was too small in the image, an example is shown in Fig. 11.

The key points-base method MediaPipe is an open-source framework, which is available for use with different programming languages and devices. The hand landmarks detection solution in Python within this framework is used to evaluate the performance. The parameter “static image mode” was set to True and the “ maximum number of hands ” was set to 1. The results in Table 3 show that the Faster-RCNN is generally more reliable for hand detection tasks than MediaPipe.

**Table 3:** Comparison of hand detection accuracy on recorded videos between Faster-RCNN and Mediapipe.

	Faster-RCNN	MediaPipe
Video 1	$100.00\%$	$93.99\%$
Video 2	$95.76\%$	$8.86\%$
Video 3	$99.82\%$	$15.74\%$
Video 4	$92.59\%$	$9.94\%$
Video 5	$91.67\%$	$12.50\%$

**Fig. 12:**
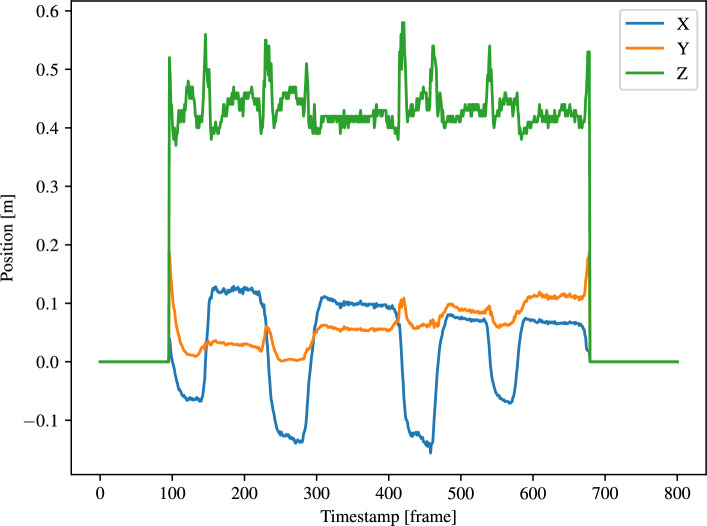
Generated hand trajectory in camera frame with Faster-RCNN.

The hand trajectories are then computed using the detection results from Faster-RCNN because it has better performance. The approach was presented in Section 3.4.1. An example of a hand trajectory is shown in Fig. 12. From this graph, the movement of the hand is more variable along the z axis (depth), as indicated by the green line. This is due to the hand moving towards and away from the camera for the multi-step pick & place task.

### Training and evaluation the object detection model

When it comes to detecting objects that are not as commonly modeled as hands, it is typically necessary to generate a custom dataset for training or fine-tuning. A total of 18,750 photorealistic images with were generated using the approach discussed in section 3.3.1 to train the YOLOv8 model for the OBB detection task. Out of these, 15,000 images are used for the training set, and 3750 images are allocated for the validation set to evaluate the performance of the model. Transfer learning approach is used with the pre-trained model YOLOv8 from Ultralytics. During training, the number of epochs was set to 20. The IoU was set as 0.7. The learning rate was set to the default value of 0.01, as this has been demonstrated to be a value for transfer learning tasks.

The Table 4 presents the object detection results for the validation set consisting of 3,750 images with a total of 21,821 instances across various classes. The overall precision and recall for detecting all classes are $98.5\%$ and $99\%$ respectively, with a mAP at $50\%$ IoU threshold of $98.7\%$ and mAP at 50–95% IoU of $47.8\%$. Each class, including GrayBox, BlueBox, Parallelogram, Cuboid, Octagon, and Star, has a high precision and recall, ranging from $96.8\%$ to $99.3\%$ and $97.4\%$ to $99.4\%$ respectively. The mAP50 for these classes varies slightly, between $98.4\%$ and $99\%$, while the mAP50-95 ranges from $43.4\%$ to $49.6\%$. The Star class has the highest mAP50-95 at $49.6\%$ and shares the highest precision and recall with the Octagon class at $99.3\%$. The Cuboid class shows a slightly lower precision compared to others at $96.8\%$. Overall, the results indicate a high level of accuracy in the object detection task for this validation set.

**Table 4:** Object detection results on validation set.

Classes	Images	Instances	Precision	Recall	mAP50	mAP50-95
all	3750	21821	$98.5\%$	$99\%$	$98.7\%$	$47.8\%$
GrayBox	3750	3633	$98.8\%$	$99.1\%$	$98.6\%$	$43.4\%$
BlueBox	3750	3670	$98.7\%$	$99.3\%$	$99\%$	$48.4\%$
Parallelogram	3750	3637	$98.3\%$	$97.4\%$	$98.4\%$	$48.3\%$
Cuboid	3750	3598	$96.8\%$	$99.4\%$	$98.4\%$	$48.4\%$
Octagon	3750	3652	$99.1\%$	$99.3\%$	$98.7\%$	$48.6\%$
Star	3750	3631	$99.3\%$	$99.3\%$	$99\%$	$49.6\%$

**Fig. 13:**
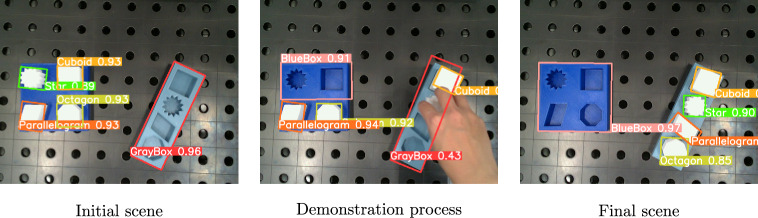
Object detection results on real world images with Faster-RCNN.

The object detection model trained on photorealistic images was evaluated on real-world images to assess its performance. Three representative images with the detection results are presented in Fig. 13. In these images, the model is able to identify and localize the object of interest within the scene. However, the occlusion and the presence of a human hand show poor performance.

To evaluate the performance of the domain adaption. Images without the presence of a human hand were considered. 971 images from the 5 recorded videos are extracted. The Table 5 presents performance for different classes, including the number of images, instances and accuracy. The model achieves impressive performance on the entire dataset, with $100\%$ accuracy indicating that all detected instances are correct. The recall of $83.3\%$ suggests that the model is capturing a significant fraction of the actual instances. The mAP50 score of $90.5\%$ indicates a high average precision at a threshold of 50. The model performs differently for each class. For instance, the GrayBox and BlueBox class exhibit lower recall values of approximately $50\%$ due to object occlusion within the box. It is worth noting that the mAP50-95 scores are relatively lower compared to the mAP50 scores, indicating potential for improvement in detecting instances within this threshold range.

**Table 5:** Object detection results on real world images.

Classes	Images	Instances	Precision	Recall	mAP50	mAP50-95
all	971	5826	$100\%$	$83.3\%$	$90.5\%$	$72.1\%$
GrayBox	971	971	$100\%$	$50.3\%$	$75.1\%$	$38.5\%$
BlueBox	971	971	$99.9\%$	$49.7\%$	$70.1\%$	$70.1\%$
Parallelogram	971	971	$100\%$	$100\%$	$99.5\%$	$73.5\%$
Cuboid	971	971	$100\%$	$100\%$	$99.5\%$	$71.4\%$
Octagon	971	971	$100\%$	$100\%$	$99.5\%$	$89.9\%$
Star	971	971	$100\%$	$100\%$	$99.5\%$	$89.3\%$

### Evaluation of depth value quality

Depth value is essential to generate the hand and the objects trajectories in Cartesian coordinates. Chen et al. discusses the common challenge of motion blur in depth sensing technology, particularly in dynamic movements with ToF sensors^42^. Depth is calculated by measuring the time it takes for a light signal to travel to the object and back to the sensor. However, movement of objects can distort the timing measurement, resulting in inaccuracies. The experimental results of calculating the depth value of hand and object show the same pattern, where the depth value of hand trajectories is generally worse than that of object trajectories. Specifically, the study notes that valid depth values for hand trajectories fall below $50\%$, whereas for objects, they exceed $70\%$. In case of invalid depth value, the depth is estimated using the method discussed in section 3.4.1. The difference between the depth value estimated by the pre-trained deep learning model and the valid depth value from the camera is less than 1cm. This evaluation demonstrates the necessity of using depth estimation techniques to ensure accurate trajectory generation and highlights the performance of the applied depth estimation method.

### Evaluation of the proposed segmentation approach

**Fig. 14:**
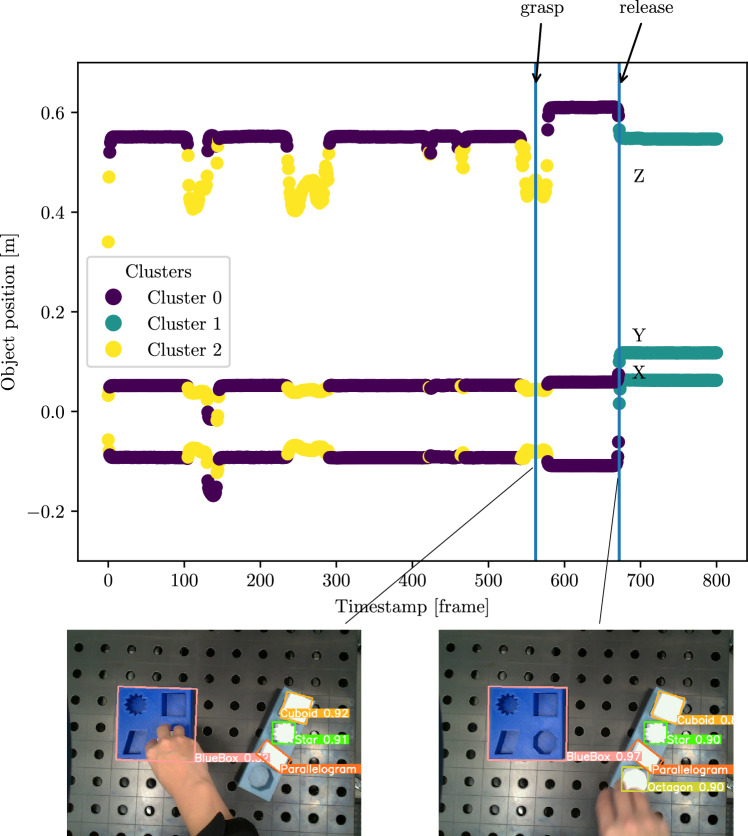
Hand trajectory segmentation results.

The hand trajectories from the recorded videos were then segmented using the Algorithm 1. They are calculated by the object trajectories and the distance between the hand and the target object. An object trajectory is outlined in Fig. 14. Each color represents a component generated by GMM. The trajectories were taken as correct segmented by inspecting the image at the identified timestamps for *grasp* and *release*. This method of verification ensures that the segmentation aligns with the actual moments when the object is manipulated. Although Table 5 shows that recall varies between object classes, the refinement step based on object states, as explained in Section 3.4.3, ensured that grasping and releasing events were recognized with 100% accuracy.

### Evaluation of the motion learning approach

**Fig. 15:**
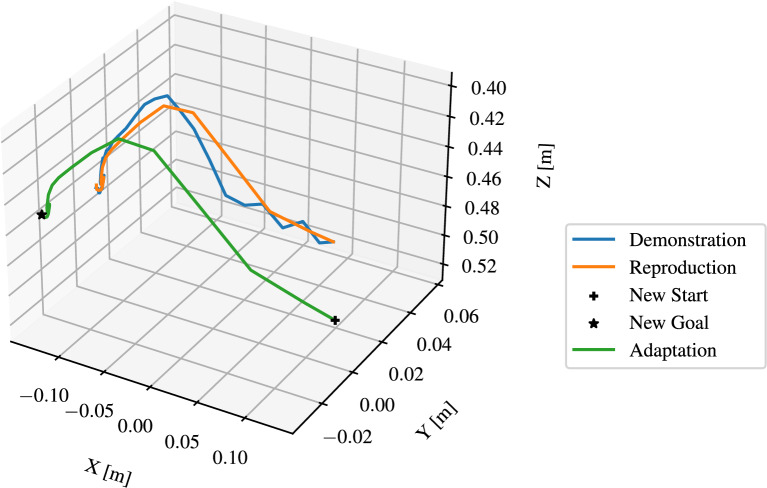
Learned and updated DMP for representing *reach* with $\lambda =0$ and $N=20$.

**Fig. 16:**
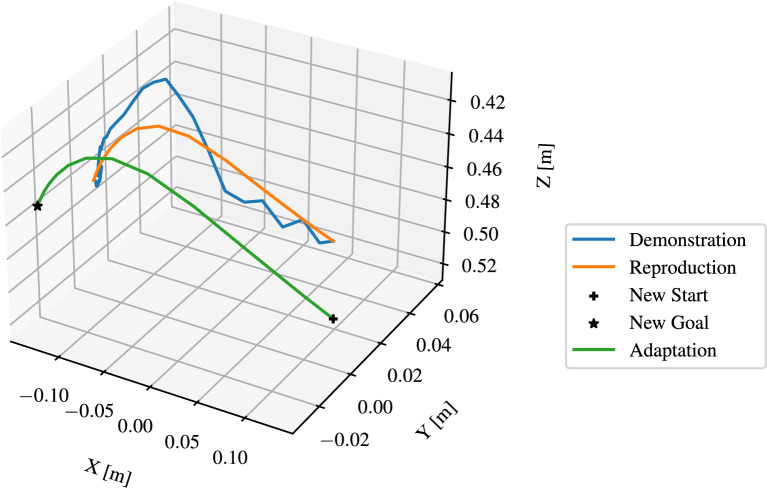
Learned and updated DMP for representing *reach* with $\lambda =0.1$ and $N=20$.

**Fig. 17:**
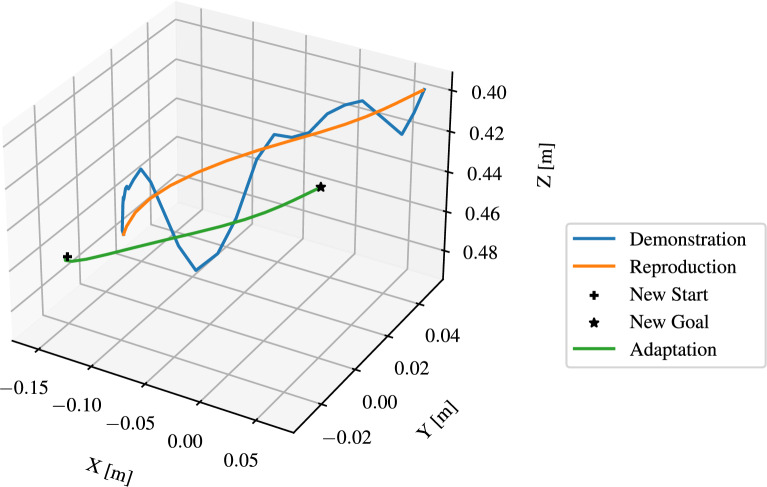
Learned and updated DMP for representing *move* with $\lambda =0.1$ and $N=20$.

As discussed in Section 3.4.2, the segmented trajectories are modeled by DMP, which represent *reach* and *move* skills. As shown in Fig. 12, the hand trajectories have irregularities, which illustrates the necessity for DMP to smooth these trajectories. Moreover, the DMP should be capable of generalizing to new start and target positions. The implementation of Fabisch^38^ in Python was used to learn the DMPs. Figure 15 illustrates the learned and updated DMP for representing the skill *reach* with regulation coefficient $\lambda$ = 0. The number of $\phi$ functions was set to *N* = 20. The “ Demonstration ” curve represents a segmented trajectory of a hand motion from the starting point to a *pre-grasp* position. The “ Reproduction ” curve shows how the DMP was learned to imitate the original hand motion by attempting to follow the demonstrated trajectory. To enhance the manipulation accuracy, the DMP was adapted to object’s position “ New Start ” and “ New Goal ”. Since $\lambda$ = 0, the noise and irregularities in the hand trajectory are encoded in the DMP, the phase approaching the goal in the figure shows the description. Figure 16 illustrates the improved DMP with $\lambda$ = 0.1, the “ Reproduction ” and “ Adaptation ” curves show that the influence of noise from the demonstration trajectory is reduced. Figure 17 shows the learned and updated DMP for representing the *move*. The differences arise from noise reduction and trajectory approximation in the DMP formulation, rather than from instability. The DMP successfully captures the demonstrated motion pattern and adapts it to new start and goal positions. In summary, the learned DMP are able to adapt learned behaviors to new situations while preserving the details of the original demonstration.

### Computational time

The evaluation was conducted on a computer running Ubuntu 20.04, equipped with an AMD Ryzen 5 3600 6-core processor (12 threads), 31.3 GiB of RAM, and a GeForce RTX 2070 Super GPU. Table 6 provides a breakdown of tasks organized into three phases: Preparation, Learning, and Execution. The time figures presented are averaged from the five recorded videos. In the preparation phase, tasks such as training data generation and fine-tuning the YOLOv8 model for object detection takes the most time, 10 hours and 2 hours, respectively. Although these tasks are time-consuming, this phase can be automated and performed independent of task learning. The learning phase starts with video recording, which takes 30 seconds, followed by converting the video to color and depth frames (70 seconds), and depth estimation, which is the most time-consuming task in the phase at 1.2 hours. After that, the system performs hand detection (18 seconds) and object detection (20 seconds). It then generates hand and object trajectories, both taking just 1 second each. This is followed by trajectory segmentation, which requires 4 seconds, and finally, the system learns dynamic movement primitives (DMPs) in less than a second. Despite the longer duration of depth estimation, the learning phase overall is highly efficient. During execution, the required trajectories can be generated in less than a second.

**Table 6:** Task breakdown with one video which contains of 597 frames.

**Phase**	**Task**	**Time**
Preparation	Training data generation	10 hours
	Fine-tuning the YOLOv8 model	2 hours
Learning	Video recording	30 seconds
	Video to color and depth frame	70 seconds
	Depth estimation	1.2 hour
	Hand detection	18 seconds
	Object detection	20 seconds
	Hand trajectory generation	1 second
	Object trajectory generation	1 second
	Trajectory segmentation	4 seconds
	Learning DMP	< 1 second
Execution	Generation DMP	< 1 second

### Evaluation on robot in simulation

This study was conducted in simulation using a Robotiq 2F-85 gripper model. The simulation setup captures geometric constraints such as opening range, while assuming sufficient gripping force for stable grasping. Real-world factors such as object density, surface roughness, and force adaptation were not explicitly considered and will be addressed in future work.

**Fig. 18:**
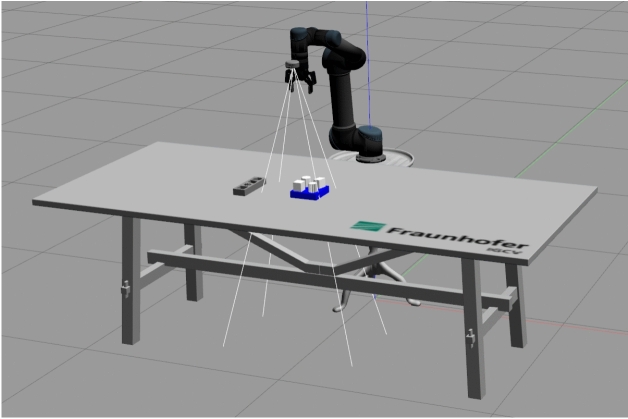
Simulation environment.

The evaluation results show a 100% success rate, with all five recorded tasks successfully executed within the ROS environment (see Fig. 18). The grasp and release positions for each object were defined by the simulation environment and the DMPs were generated accordingly. This performance highlights the effectiveness of the one-shot visual imitation learning framework, which demonstrates robust learning of human actions. In addition, the system shows adaptability to changes in object positions, further reinforcing its effectiveness in dynamic environments.

## Discussion

The results highlight the potential of one-shot visual imitation learning to simplify and accelerate robot programming in industrial contexts. The modular framework proved effective in reproducing multi-step pick & place tasks from a single demonstration. The modularity also enabled the integration of pre-trained models and CAD-based synthetic data generation, making one-shot learning feasible despite the lack of large annotated datasets in manufacturing.

At the same time, several limitations must be acknowledged. For hand detection, Faster-RCNN was reliable in controlled conditions but struggled when the hand appeared small in the image. For object detection, synthetic data generation from CAD models proved effective in training, yet domain adaptation is still necessary to achieve high accuracy in real-world environments. Advanced domain randomization or hybrid datasets combining synthetic and real images could improve robustness.

The segmentation algorithm effectively identified grasp and release events, and object-state refinement ensured that these critical transitions were recognized with 100% accuracy despite imperfect frame-level recall. However, trajectory generation using DMPs remains unoptimized with respect to efficiency and collision avoidance. Furthermore, the computational bottleneck in depth estimation indicates that faster and more robust depth pipelines are required for real-time applications.

## Conclusion

This paper presented a one-shot visual imitation learning framework that enables robots to learn multi-step pick & place tasks from a single video demonstration. The framework demonstrates significant potential for reducing the data requirements and complexity of robot programming, making it particularly valuable for manufacturing contexts where flexibility and efficiency are essential. While the current evaluation was conducted in simulation with predefined gripping parameters, the results provide a solid foundation for future real-world studies. Future work will focus on enhancing object detection robustness, optimizing trajectory generation, improving computational efficiency, and extending evaluation to real hardware and diverse gripper types.

In summary, the proposed approach represents a significant step toward intuitive, flexible, and efficient robot programming by non-experts, while its modular design ensures adaptability to a broad range of industrial applications.

## Data Availability

The datasets generated and/or analysed during the current study are available in the Zenodo repository, https://doi.org/10.5281/zenodo.15426315
